# Adaptive kernel selection network with attention constraint for surgical instrument classification

**DOI:** 10.1007/s00521-021-06368-x

**Published:** 2021-09-13

**Authors:** Yaqing Hou, Wenkai Zhang, Qian Liu, Hongwei Ge, Jun Meng, Qiang Zhang, Xiaopeng Wei

**Affiliations:** School of Computer Science and Technology, Dalian University of Technology, Dalian, China

**Keywords:** Health care, Deep learning, Fine-grained classification, Attention mechanism

## Abstract

Computer vision (CV) technologies are assisting the health care industry in many respects, i.e., disease diagnosis. However, as a pivotal procedure before and after surgery, the inventory work of surgical instruments has not been researched with the CV-powered technologies. To reduce the risk and hazard of surgical tools’ loss, we propose a study of systematic surgical instrument classification and introduce a novel attention-based deep neural network called SKA-ResNet which is mainly composed of: (*a*) A feature extractor with selective kernel attention module to automatically adjust the receptive fields of neurons and enhance the learnt expression and (*b*) A multi-scale regularizer with KL-divergence as the constraint to exploit the relationships between feature maps. Our method is easily trained end-to-end in only one stage with few additional calculation burdens. Moreover, to facilitate our study, we create a new surgical instrument dataset called SID19 (with 19 kinds of surgical tools consisting of 3800 images) for the first time. Experimental results show the superiority of SKA-ResNet for the classification of surgical tools on SID19 when compared with state-of-the-art models. The classification accuracy of our method reaches up to 97.703%, which is well supportive for the inventory and recognition study of surgical tools. Also, our method can achieve state-of-the-art performance on four challenging fine-grained visual classification datasets.

## Introduction

The health care sector has long been an early adopter and benefited greatly from technological advances. In recent years, artificial intelligence (AI) technologies, i.e., deep neural networks, play a key role in many health-related realms, including disease prediction [17] and diagnosis [19], intelligent robot-assisted surgery [16], health monitoring [46], the development of new medical procedures [1], etc.

Computer vision (CV), as one of the most successful research directions in the field of AI, has achieved remarkable breakthroughs in the health care industry, helping medical professionals in saving their valuable time on basic tasks while also saving patients’ life. The focus of CV in health care has been placed on solving various medical tasks by processing different types of medical/pathological images. For example, in the early recurrence prediction of hepatocellular carcinoma [50], radiomics features are extracted from arterial and portal venous-phase CT images for evaluating the preoperative clinical factors. Besides, medical image processing has also been applied to solve different medical tasks, including computer-aided detection (CADe) in radiological diagnoses [34], prostate image analysis based on 3D image segmentation [30], 3D MRI brain coronal slices image registration [3], detection of critical findings in head CT scans [7], etc. These studies or designs in the field of CV have mainly been considered as auxiliary tools for doctors in numerable analysis or operations, thereby providing instructions to obtain a higher precision on diagnosis, prediction, screening, tracking and so on.

Despite the remarkable success of CV on auxiliary medical diagnosis, recent studies have also actively ventured into other emerging application domains in the health care sector, for example, the robot-assisted surgery based on a 3D camera [15], rehabilitation training based on vision reconstruction for people with visual impairment [6], health monitoring on patients for disease prediction and prevention [33], etc. Among these studies, relevant research works on medical instrument images, i.e., the surgical instruments, which are the most important tools in the procedure of surgery, have less been explored. Notably, the inventory work of the surgical instruments before and after surgeries is of great importance for medical safety. At present, the surgical instrument inventory work is mostly carried out by the professional medical staff. Nevertheless, mistakes occur inevitably due to the negligence or fatigue of human beings. In 2020, the Australian Productivity Commission released a number of medical records that indicate the nationwide medical malpractice has taken off, and 430,000 patients have suffered. Medical malpractices related to medical devices are even more noticeable among these patients. As the Daily Mail goes, the Bungling surgeons left medical instruments inside at least 23 patients who were poisoned, infected, or injured in hospital in just a year. Therefore, it is much of significance to ensure the reliability of inventory work of surgical instruments.

In the surgical instrument inventory work, the medical staff is mainly responsible for checking the type and quantity of surgical instruments. And the identification of surgical instruments is one of the focuses of the verification work. Taking this cue, this paper takes a series of research works on surgical instrument inventory work, with the aim to accurately identify surgical instruments before and after surgery. This work not only saves human resources, but also quickly identifies whether surgical instruments are missed, which is beneficial in preventing secondary infections or fatal medical accidents. At present, medical image studies regarding surgical instruments have mainly focused on surgical tool detection, segmentation and tracking during surgery, so that doctor assistants take a more accurate grasp of the operation process [11]. To the best of our knowledge, the surgical instrument recognition work in this paper serves as the first attempt to identify and classify a surgical instrument kit for the inventory work of surgery.

**Fig. 1:**
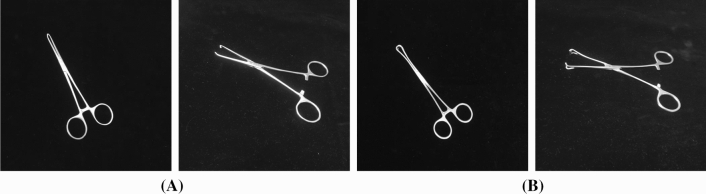
Two distinct forceps categories from the proposed SID19 dataset. **a** Alice forceps with different states, views and angles; **b** Appendix forceps with different states, views and angles

To support the work, a novel surgical instrument dataset is built. As reported in statistics from the University of Rochester Medical Center, the most common surgical operations in the USA mainly include appendectomy, breast biopsy, carotid endarterectomy, cataract surgery, etc. Accordingly, this work considers 19 categories of surgical instruments from surgical kits of appendix resection, cholecystectomy surgical and cesarean section (including Alice forceps, hemostatic forceps of different sizes, oval forceps, suction head, four kinds of hooks, needle holders, cloth forceps, long and short tooth forceps, thread scissors, tissue scissors, intestinal plate, etc.) as the raw materials to create our surgical instrument dataset (labeled as SID19). Notably, in the proposed SID19, there exist certain surgical tools that belong to the fine-grained classes, which possess the very subtle differences that are intractable to distinguish from one another. Among them, surgical forceps and surgical scissors both contain several sub-categories, namely fine-grained classes. In the proposed SID19, categories of surgical forceps include Alice forceps, hemostatic forceps of different sizes, oval forceps, long and short tooth forceps, and cloth forceps. Categories of surgical scissors include thread scissors, tissue scissors, etc. Objects in these sub-categories usually share large intra-class and small inter-class variances, introducing difficulties for identification task. For example, Fig. 1 displays two forceps categories: Alice forceps and appendix forceps with different states, views and angles. Alice forceps and appendix forceps have a tiny difference in their fore-end, where the fore-end of appendix forceps is much rounder than that of Alice forceps. Therefore, different from the common natural image classification, the presented surgical instrument classification task reveals unique characteristics of fine-grained visual classification (FGVC), thus bring additional difficulties.

To tackle the FGVC problem, recent works for FGVC mainly focus on weakly supervised learning, which can be roughly grouped into two categories: attention-based methods to strengthen the intermediate feature maps and other methods to exploit the relationships between feature maps. However, the whole framework is fairly complex due to the additional attention network for the first group and the design of regularizer for the other group is complicated too. Additionally, leveraging the strengthened intermediate feature maps extracted from different stages to explore the relationships of feature maps is rarely exploited. In this paper, we propose a novel fine-grained visual classification framework named SKA-ResNet to explore the efficacy of surgical instrument classification. Our method involves two novel components: a feature extractor with stacked standard residual blocks with selective kernel attention (SKA) module to boost the intermediate feature maps and a multi-scale regularizer to explore the relationships of strengthened intermediate feature maps.

In the feature extraction stage, we propose to embed the SKA module into the end of standard residual block forming a new block called SKA block. As the core of our feature extractor, SKA module is capable of generating special attentions with different receptive fields information to strengthen the intermediate feature maps, which can be described into three different stages, including *Divide*, *Fuse* and *Aggregation*. Particularly, an attention factor with special information is generated by leveraging adaptive kernel selection and SGE attention mechanism, so that the informative expression can be strengthened efficiently. Besides, we introduce a multi-scale regularizer to explore the relationships of different scale feature maps boosted by SKA module. The mid-level and high-level feature maps extracted from different stages are strengthened through a CBAM-layer similar to the CBAM module [42]. Then, the enhanced feature maps and the last outputs of the feature extractor are concatenated before classification. Meanwhile, the relationships of the enhanced feature maps are constrained by a regularizer that matches the prediction distribution of the mid-level features to the high-level ones with KL-divergence. The experimental results demonstrate that our method achieves the best performance on SID19 by around 97.703%, which is feasible for assisted decision-making in inventory work. Moreover, our method outperforms the state-of-the-art models on four standard benchmark datasets.

Main contributions of the work are summarized as follows:

1. Considering the medical accidents about loss of surgical instruments during surgeries, surgical instrument classification is proposed for the first time to assist medical staff in inventory work for reducing the medical accidents risk.
2. To explore the work of surgical instrument classification, we adopt the surgical kits corresponding to three most common surgeries (appendectomy, cholecystectomy and cesarean section) as origin materials to create a dataset, SID19, wherein 19 kinds of surgical tools consisting of 3800 images are collected.
3. A novel attention-based model called SKA-ResNet is proposed to explore the classification work. The network can capture subtle differences among fine-grained classes by embedding the selective kernel attention module into feature extractor. Further, a multi-scale regularizer is proposed to boost the classification.
4. Results show that our method achieves a high accuracy of around 97.703% on SID19, which is superior to existing methods. Also, it achieves superior performance on four challenging fine-grained visual classification datasets when compared to the state-of-the-arts.

The rest of this paper is organized as follows. Section 2 introduces background of the work. Section 3 describes the proposed SKA-ResNet in detail. In Sect. 4, we describe the details of our proposed SID19 dataset. Section 5 shows the experimental settings and results on SID19 and other datasets. Section 6 concludes the paper.

## Background

### CV applications in health care

Computer vision, as one of the most successfully applied technologies in AI, has been introduced into a wide range of fields to solve specific tasks. Nowadays, various CV-powered technologies are assisting the health care industry in all respects. As a result, medical professionals get a better knowledge about diseases so that they make a sound judgment or even save patients’ lives.

Today’s health care industry strongly relies on precise diagnostics provided by medical imaging, which works with data obtained by different diagnostic technologies including X-ray, computed tomography (CT), magnetic resonance imaging (MRI), etc. Based on heterogeneous pathologic images, medical image analysis has focused on disease prevention, prediction, detection, diagnosis, screening and so on. For example, in the early diagnose of chronic obstructive pulmonary disease, Filho et al. [8] propose to utilize information from lung CT images to identify and classify lung diseases with the automatic feature extractor. Moreover, to detect the stages of cancer if affected, Sekaran et al. [36] utilize CNN to predict the cancer images of the pancreas, which is embedded with the model of Gaussian mixture model with EM algorithm to identify the essential features from the CT Scan. More recently, under the screening work of the coronavirus disease (COVID-19), Wang et al. [40] extract COVID-19’s specific graphical features and provide a clinical diagnosis ahead of the pathogenic test derived from the radiographical changes in CT images, thus saving critical time for disease control. In the detection of COVID-19, Apostolopoulos et al. [2] suggest that the state-of-the-art CNN architectures proposed over the recent years for medical image classification with transfer learning are successful in extracting significant biomarkers related to the COVID-19 disease based on X-ray imaging.

Besides the success of CV in medical pathologic analysis, new applications in the health care sector have also emerged. For example, under health monitoring, Suo et al. [39] build a personalized time fusion framework to predict patients’ risk of developing certain diseases by monitoring changes in patient visit time. In computer-assisted surgery, Pakhomov et al. [31] focus on binary instrument segmentation by leveraging deep residual learning and dilated convolutions. Moreover, Zhao et al. [47] propose a visual tracking approach using the CNN with a spatial transformer network and a spatiotemporal context learning algorithm for the process of tool tracking frame by frame, which is devoted to enhancing the context-awareness of surgeons in the operating room. Sanchez-Garcia et al. [35] present a new CNN-based fusion approach to build a schematic representation of indoor environments for simulated phosgene images, which aims to train and partially recover the retinal stimulation of visually impaired people in rehabilitation training.

In this paper, we propose to take a series of study centered around the identification of surgical instruments before and after surgery. The study aims to save human resources and reduce the risk of secondary infections or fatal medical accidents incurred by the loss of surgical instruments. The proposed work is carried out upon a newly designed surgical instrument dataset, which is quite different from those relying on pathological images. Additionally, in comparison with tool detection, segmentation and tracking relying on surgery videos in computer-assisted surgery, our study works before and after surgery for the inventory of surgical tools.

### Fine-grained visual classification

Research works for FGVC tasks mainly proceed along two dimensions, namely strongly supervised learning and weakly supervised learning. Specifically, strongly supervised learning methods add the object bounding boxes, part annotation information and image level labels to the training network for learning specific discriminative location information of the targets [14, 24, 45]. Nevertheless, this sort of methods suffer as (a) a huge amount of human resources are demanded to label the original images, and (b) the information marked by humans is not accurate sometimes. On the contrary, weakly supervised learning networks are only given the categories of images for classification.

As the most frequently used method in CV research works, attention mechanisms have been widely employed in various classification, detection and segmentation tasks, especially in weakly supervised FGVC tasks. According to attention mechanisms, the informative features are strengthened and the less useful ones are suppressed, simultaneously. Lots of lightweight attention modules are introduced in recent years. For example, a high-efficiency, lightweight gating mechanism is introduced in SENet [13] to strengthen the intermediate feature maps via channel-wise importance. Beyond channel dimension, BAM [32] and CBAM [42] generate attention maps along spatial and channel dimensions for adaptive feature reinforcement. Based on group convolution, SGENet [21] proposes a novel spatial group-wise enhanced attention, which focuses on learning different semantic sub-feature maps of each group, intentionally self-enhancing its spatial distribution. Except for spatial and channel dimensions, SKNet [22] firstly suggests to explicitly exploring the adaptive receptive field (RF) size of neurons by introducing a dynamic kernel selection mechanism which is constructed by multi-branch convolutions based on different kernels. All the above attention mechanisms constitute lightweight attention modules, which can be embedded into majority backbone networks, promoting the performance of networks. Based on attention mechanism, some methods [9, 38, 44] construct the additional attention networks for FGVC problem. Although these methods can obtain excellent performance, the architectures of these methods are complicated due to the additional attention networks when comparing with lightweight attention modules.

On the other hand, there are other weakly supervised models introduced in FGVC for feature relationship learning. Methods based on high-order statistics are proposed in visual classification, especially for solving the FGVC problem. Specifically, bilinear CNN (BCNN) [25] performs element-wise square root normalization followed by $\ell _{2}-$ normalization for bilinear features, achieving impressive performance. Compact bilinear CNN [10] proposes two compact bilinear representations with the same discriminative power as the full bilinear representations but with only a few dimensions compared with bilinear features. By the same token, the core of iSQRT-COV [20] is a meta-layer with loop-embedded directed graph structure, specifically designed for ensuring both convergence of Newton-Schulz iteration and performance of global covariance pooling networks. Other methods propose to exploit relationships of different scale feature maps. Cross-x learning [29] introduces an approach to exploit the relationships between different images and different network layers for robust multi-scale feature learning.

However, our method differs from previous works in two aspects: First, embedding adaptive kernel selection mechanism with SGE attentions, our SKA module can strengthen the expression of discriminative regions automatically which is lightweight and efficient. Second, we utilize a multi-scale regularizer to exploit the relationships between the strengthened feature maps for robust performance. In particular, the two parts are complementary in our approach. On the one hand, the feature extraction network based on the attention mechanism can generate feature maps with rich semantic information for multi-scale learning and fundamentally improve the performance of multi-scale feature learning; on the other hand, multi-scale feature learning uses attention-enhanced feature maps combining with constraint conditions to guide the generation of feature maps in the feature extraction stage.

**Fig. 2:**
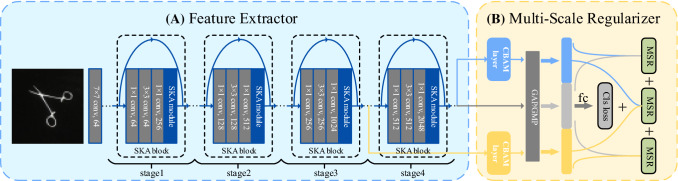
The framework of our SKA-ResNet consists of two parts.** A**: A feature extractor with SKA module embedded to extract high expression feature maps;** B**: A multi-scale regularizer taking relationships between feature maps as a constraint to exploit multi-scale learning

## Method

In this section, the detailed architecture of SKA-ResNet is delineated. As depicted in Fig. 2, the whole network is composed of two main components: (1) A novel feature extractor consisting of stacked standard residual blocks with Selective Kernel Attention (SKA) modules that extracts informative feature maps for discriminative regions without additional attention networks. (2) A multi-scale regularizer taking relationships between feature maps and images as a constraint that learns the relationships of different fine-grained categories. Different from existing attention-based methods focusing on additional attention networks, our method embeds lightweight SKA modules into the standard residual blocks in a scattered way. Furthermore, the relationships between different feature maps and images are exploited by a multi-scale regularizer for robust fine-grained feature representation. And the entire network is trained end-to-end simply relying on image level labels.

### SKA module

**Fig. 3:**
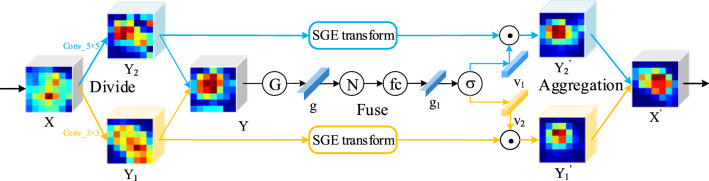
The detailed procedure of our SKA module with two selective kernel branches is illustrated

In the stage of feature extraction, we introduce a novel and lightweight SKA module as depicted in Fig. 3. The feature extractor can localize the discriminative regions and strengthen the corresponding feature maps automatically by embedding the SKA modules into stacked standard residual blocks of ResNet. Operations in the proposed SKA module are summarized into *Divide*, *Fuse* and *Aggregation*. As shown in Fig. 3, we take the SKA module with a two-branch case as an example for the detailed illustration.

**Divide** In the *Divide* stage, given intermediate feature map $X \in R^{W\times H\times C}$, it is first sent into two different convolutional layers to generate two feature maps with different semantic information. The convolution layers are grouped with convolutions, Batch Normalization and ReLU function in sequence. Specifically, the two convolution layers are conducted by a $3 \times 3$ kernel size and a $5 \times 5$ kernel size, respectively. The two obtained feature maps are expressed as $Y_1 \in R^{W\times H\times C}$ and $Y_2 \in R^{W\times H\times C}$. Note that, the size of the two obtained feature maps is the same as the original feature map *X*. The procedure can be summarized as:

1 $$\begin{aligned} \mathbf {Y_1}= & {} \delta \left( {{\mathcal {B}}}{{\mathcal {N}}}\left( {Conv}{3\times 3}\left( {\mathbf {X}}\right) \right) \right) \in {\mathbb {R}}^{W \times H \times C}, \end{aligned}$$

2 $$\begin{aligned} \mathbf {Y_2}= & {} \delta \left( {{\mathcal {B}}}{{\mathcal {N}}}\left( {Conv}{5\times 5}\left( {\mathbf {X}}\right) \right) \right) \in {\mathbb {R}}^{W \times H \times C}, \end{aligned}$$

where $\delta$, ${{\mathcal {B}}}{{\mathcal {N}}}$ and *Conv* refer to ReLU function, Batch Normalization and convolutions, respectively.

**Fuse** To enable neurons to adaptively adjust their RF sizes according to the stimulus content, an element-wise summation gate is adopted to integrate different information from the two branches. We generate the mixed feature map $Y \in R^{W\times H\times C}$ by a summation gate. Then, the global information of *Y* is generated by utilizing global average pooling, which is noted as $g \in R^{C}$. To prevent the biased magnitude of coefficients between various samples, we employ a normalization in *g* over the channel. Further, the obtained global feature vector is sent to a *fc* layer, which is conducted with convolutional layers with $1 \times 1$ kernel size, Batch Normalization and ReLU function, meanwhile reducing the dimension of *g* for better efficiency. The obtained compact feature vector is expressed as $g_1 \in R^{C_1}$, where $C_1$ is the dimension after reduction in dimensionality by $1\times 1$ convolutions. The relationship between *C* and $C_1$ is controlled by a parameter defined as *r*, where $C_1=max(C/r)$ and the minimum value of $C_1$ is not less than 32. Specially, we embed SGE module into the different branches to generate ${Y}_{1}^{'}$ and ${Y}_{2}^{'}$ with spatial group-wise enhanced attentions, respectively. The procedure can be summarized as:

3 $$\begin{aligned} \mathbf {g_1} =   {fc}\left( {\mathcal {N}} \left( {\mathcal {F}}_{gp} \left( \mathbf {Y_1} + \mathbf {Y_2}\right) \right) \right) \in {\mathbb {R}}^{C_1}, \end{aligned}$$

4 $$\begin{aligned} \mathbf {{Y}_{1}^{'}} =   {SGE}\left( \mathbf {Y_1}\right) \in {\mathbb {R}}^{W \times H \times C}, \mathbf {{Y}_{2}^{'}}={SGE}\left( \mathbf {Y_2}\right) \in {\mathbb {R}}^{W \times H \times C}, \end{aligned}$$

where *fc*, ${\mathcal {N}}$ and ${\mathcal {F}}_{gp}\left( {\cdot }\right)$ refer to the above *fc* layer, Normalization and Global Average Pooling. And *SGE* refers to the operations of SGE module.

**Aggregation** A softmax operator is applied to the global feature vector $g_1$ to select different RFs of information, which can be regarded as a soft attention mechanism. Through the operation, we obtain two different informative feature vector $v_1$ and $v_2$ corresponding to $Y_1$ and $Y_2$, respectively. For the two generated weights vector $v_1$ and $v_2$, we generate the strengthened feature maps $A \in R^{W\times H\times C}$ and $B \in R^{W\times H\times C}$ by employing $v_1$ to scale ${Y}_{1}^{'}$ and $v_2$ to scale ${Y}_{2}^{'}$. The final feature map $X^{'}$ is obtained by summation of the result of two branches. The procedure can be summarized as:

5 $$\begin{aligned} \left[ \mathbf {{v}_{1}}, \mathbf {{v}_{2}}\right] =  {softmax}\left( \mathbf {g_1}\right) , \end{aligned}$$

6 $$\begin{aligned} \mathbf {{X}^{'}} =  \mathbf {{v}_{1}} \cdot \mathbf {{Y}_{1}^{'}} + \mathbf {{v}_{2}} \cdot \mathbf {{Y}_{2}^{'}}, \end{aligned}$$

where *softmax* refers to the *softmax* function.

**Fig. 4:**
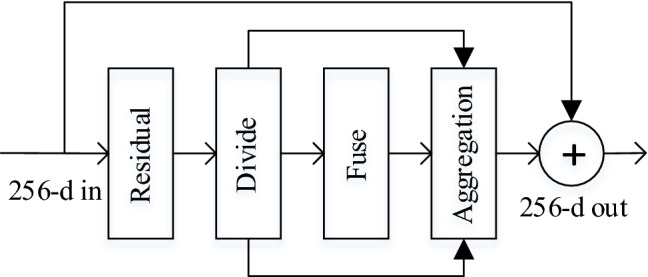
The example standard residual block with SKA module called SKA block, in which “Residual” refers a sequence of convolutional layers in standard residual block of ResNet

As shown in Fig. 4, the standard residual block with our proposed SKA module is exhibited. The proposed SKA module is lightweight without introducing too many volumes of calculations and parameters, so that it can be easily embedded into any mainstream backbone network. Further, it is of high-efficiency in learning informative feature maps for fine-grained visual classification tasks. We employ SKA module to the standard residual block of ResNet for structuring a novel and efficient block that constitutes the core of our feature extractor. By the above feature extractor, we can extract informative feature maps.

### Multi-scale regularizer

Multi-scale learning (MSL) has been shown to be useful for numerable visual tasks [5, 18, 27, 28] . Mid-level feature maps usually bear more precise location information, while the high-level ones take more discriminative semantic information. Thus, we apply the simple idea that multi-scale feature maps extracted from different layers are combined to form a pyramid structure for prediction like FPN [26] does. However, the relationships between different feature maps are rarely exploited. Further, the combination of attention module and feature relationship learning is not explored.

Under the above feature extractor, we first extract feature maps from mid-level layers and high-level layers. As shown in Fig. 2, let ${\mathbf {F}}^{3}$ and ${\mathbf {F}}^{4}$ be the feature maps of different layers (3 and 4 refer to *stage*3 and *stage*4 of ResNet depicted in Fig. 2), which can be defined as:

7 $$\begin{aligned} {\mathbf {F}}^{3} = \left\{ {\mathbf {f}}_{c}^{3}\right\} _{c=1}^{C_3} \in {\mathbb {R}}^{C_3 \times H_3 \times W_3}, {\mathbf {F}}^{4}=\left\{ {\mathbf {f}}_{c}^{4}\right\} _{c=1}^{C_4} \in {\mathbb {R}}^{C_4 \times H_4 \times W_4}, \end{aligned}$$

where *C* is the number of feature channels and $H \times W$ is the spatial size of the feature map. Then, ${\mathbf {F}}^{3}$ and ${\mathbf {F}}^{4}$ are fed to a CBAM layer [42] to strengthen the semantic information. The procedure can be summarized as:

8 $$\begin{aligned} \widetilde{{\mathbf {F}}} =  \sigma \left( \textit{MLP}\left( \textit{AvgPool}\left( {\varvec{F}}^{n}\right) \right) +\textit{MLP}\left( \textit{MaxPool}\left( {\varvec{F}}^{n}\right) \right) \right) , \end{aligned}$$

9 $$\begin{aligned} \hat{{\mathbf {F}}}^{n}=  \sigma \left( f^{7 \times 7}([\textit{AvgPool}(\widetilde{{\mathbf {F}}}); \textit{MaxPool}(\widetilde{{\mathbf {F}}})])\right) , \end{aligned}$$

where $\sigma$, $\textit{AvgPool}$, $\textit{MaxPool}$ and $\textit{MLP}$ refer to Sigmoid, Global Average Pooling, Global Max Pooling and Multi-layer Perceptron. And $f^{7 \times 7}$ refers to a convolutional layer with a kernel size $7 \times 7$. ${\hat{\mathbf {F}}}^{n}$ is the output of CBAM layer. Especially, ${\hat{\mathbf {F}}}^{3}$ and ${\hat{\mathbf {F}}}^{4}$ are the corresponding outputs of ${\mathbf {F}}^{3}$ and ${\mathbf {F}}^{4}$.

Afterward, three prediction distributions, ${\mathbf {P}}^{3}$, ${\mathbf {P}}^{4}$ and ${\mathbf {P}}$, are obtained from the last full connection layer and a SoftMax function. Note that, ${\mathbf {P}}$ is corresponding to ${\mathbf {F}}^{4}$, which is the output feature maps of the original feature extractor. To explore the relationships between feature maps extracted from *stage*3 and *stage*4, we propose a regularizer to match different prediction distributions. In terms of implementation, KL-divergence is applied in this paper as a constraint, which can be expressed as:

10 $$\begin{aligned} \begin{aligned} {\mathcal {L}}_{msl}\left( {\mathbf {P}}^{4}, {\mathbf {P}}^{3}\right)&={\mathcal {K}} {\mathcal {L}}\left( {\mathbf {P}}^{4} \Vert {\mathbf {P}}^{3}\right) =\frac{1}{N} \sum _{i=1}^{N} \sum _{j=1}^{C} p_{i j}^{4} \log \frac{p_{i j}^{4}}{p_{i j}^{3}} , \end{aligned} \end{aligned}$$

where *C* refers to the class number, and *N* donates the number of a mini-batch. $p_{i j}$ refers to the probability value of the i-th sample belonging to the j-th category. KL-divergence suggests ${\mathbf {P}}^{3}$ to match with ${\mathbf {P}}^{4}$ by minimizing the loss function ${\mathcal {L}}_{msl}$. A similar regularizer can be added to constrain ${\mathbf {P}}^{3}$, ${\mathbf {P}}$ and ${\mathbf {P}}$, ${\mathbf {P}}^{4}$ as well.

### Optimization

Given prediction distributions ${\mathbf {P}}^{3}$, ${\mathbf {P}}^{4}$ and ${\mathbf {P}}$, the loss function for classification can be expressed as:

11 $$\begin{aligned} {\mathcal {L}}_{cls}=\sum _{i=1}^{N} \sum _{j=1}^{C} {\mathcal {L}}_{c}\left( {\mathbf {P}}_{ij}^{3}+{\mathbf {P}}_{ij}^{4}+{\mathbf {P}}_{ij}, {\mathbf {P}}^{*}\right) , \end{aligned}$$

where ${\mathcal {L}}_{c}$ donates the cross-entropy loss and ${\mathbf {P}}^{*}$ is the ground-truth label vector. *C* refers to the class number, and *N* donates the number of a mini-batch. Finally, the whole model is optimized by the loss function defined as:

12 $$\begin{aligned} {\mathcal {L}}_{total}={\mathcal {L}}_{\text{ cls }}+\alpha {\mathcal {L}}_{msl}, \end{aligned}$$

where $\alpha$ is a hyper-parameter to balance the contribution to different parts. In our settings, $\alpha =1$.

**Table 1:** The four columns refer to ResNet50 backbone, SGE-ResNet50, SK-ResNet50 and the proposed SKA-ResNet50, respectively

Stage	Output	ResNet50	SGE-ResNet50	SK-ResNet50	SKA-ResNet50
conv1	$112 \times 112$	$7 \times 7$, 64, stride 2			
conv2	$56 \times 56$	$7 \times 7$ max pool, stride 2			
		$$\left[ \begin{array}{lr} 1\times 1, 64 \\ 3\times 3, 64 \\ 1\times 1, 256 \end{array} \right] \times 3$$	$$\left[ \begin{array}{lr} 1\times 1, 64 \\ 3\times 3, 64 \\ 1\times 1, 256 \\ SGE\,module \end{array} \right] \times 3$$	$$\left[ \begin{array}{lr} 1\times 1, 64 \\ 3\times 3, 64 \\ 1\times 1, 256 \\ SK\,module \end{array} \right] \times 3$$	$$\left[ \begin{array}{lr} 1\times 1, 64 \\ 3\times 3, 64 \\ 1\times 1, 256 \\ SKA\,module \end{array} \right] \times 3$$
conv3	$28 \times 28$	$$\left[ \begin{array}{lr} 1\times 1, 128 \\ 3\times 3, 128 \\ 1\times 1, 512 \end{array} \right] \times 4$$	$$\left[ \begin{array}{lr} 1\times 1, 128 \\ 3\times 3, 128 \\ 1\times 1, 512 \\ SGE\,module \end{array} \right] \times 4$$	$$\left[ \begin{array}{lr} 1\times 1, 128 \\ 3\times 3, 128 \\ 1\times 1, 512 \\ SK\,module \end{array} \right] \times 4$$	$$\left[ \begin{array}{lr} 1\times 1, 128 \\ 3\times 3, 128 \\ 1\times 1, 512 \\ SKA\,module \end{array} \right] \times 4$$
conv4	$14 \times 14$	$$\left[ \begin{array}{lr} 1\times 1, 256 \\ 3\times 3, 256 \\ 1\times 1, 1024 \end{array} \right] \times 6$$	$$\left[ \begin{array}{lr} 1\times 1, 256 \\ 3\times 3, 256 \\ 1\times 1, 1024 \\ SGE\,module \end{array} \right] \times 6$$	$$\left[ \begin{array}{lr} 1\times 1, 256 \\ 3\times 3, 256 \\ 1\times 1, 1024 \\ SK\,module \end{array} \right] \times 6$$	$$\left[ \begin{array}{lr} 1\times 1, 256 \\ 3\times 3, 256 \\ 1\times 1, 1024 \\ SKA\,module \end{array} \right] \times 6$$
conv5	$7 \times 7$	$$\left[ \begin{array}{lr} 1\times 1, 512 \\ 3\times 3, 512 \\ 1\times 1, 2048 \end{array} \right] \times 3$$	$$\left[ \begin{array}{lr} 1\times 1, 512 \\ 3\times 3, 512 \\ 1\times 1, 2048 \\ SGE\,module \end{array} \right] \times 3$$	$$\left[ \begin{array}{lr} 1\times 1, 512 \\ 3\times 3, 512 \\ 1\times 1, 2048 \\ SK\,module \end{array} \right] \times 3$$	$$\left[ \begin{array}{lr} 1\times 1, 512 \\ 3\times 3, 512 \\ 1\times 1, 2048 \\ SKA\,module \end{array} \right] \times 3$$
	$1 \times 1$	$7 \times 7$ global average pool, 1000-d *fc*, softmax			
#Params.		25.56M	25.56M	26.15M	26.15M
GFLOPs		4.122	4.127	4.185	4.195

Inside the brackets is the general shape of a residual block, including filter sizes and feature dimensionalities. The number of stacked blocks on each stage is presented outside the brackets. All modules are embedded into the end of the standard residual block. #Params. denotes the number of parameters and GFLOPs represents the number of multiply-adds

### Network architecture

Using standard residual blocks with SKA modules, the overall feature extractor architecture of SKA-ResNet50 is listed in Table 1. Besides, as the backbone network of the proposed method and two existing excellent methods based on lightweight attention modules, architectures of the other three models, ResNet50, SGE-ResNet50 and SK-ResNet50, are displayed in Table 1, as well. Similar with ResNet, the proposed SKA-ResNet mainly consists of a stack of repeated residual blocks termed as “SKA blocks.” Each SKA block is composed of a sequence of convolution layers and a lightweight SKA module. Generally, the proposed SKA module can be regarded as an independent unit. We obtain the SKA block by adding the unit to the end of sequence operations within the standard residual block. Due to the high-efficiency design of SKA module, SKA-ResNet50 only leads to 2% increase in the number of parameters and 1.8% increase in computational cost, compared with ResNet50. Further, combining adaptive RFs with SGE attention mechanism, SKA-ResNet50 yet introduces the increase in parameters compared with SK-ResNet50, because there are no convolution layers in the SGE attention mechanism. Meanwhile, it brings only a little bit of an increase in computational cost.

In SKA block, there is an important hyper-parameter called *cardinality* which dominates the number of group convolutions in the SGE attention mechanism and a reduction ratio *r* that controls the number of *fc* layer parameters in the *Fuse* stage. In the integral structure of the network, we adopt a similar topological architecture with ResNet. Especially, Table 1 shows the structure of a 50-layer SKA-ResNet which has four stages with $\left\{ 3,4,6,3\right\}$ SKA blocks, respectively. By varying the number of SKA blocks in each stage, one can obtain different architectures. In the study, we adopt SKA-ResNet50 as the primary architecture by default.

**Fig. 5:**
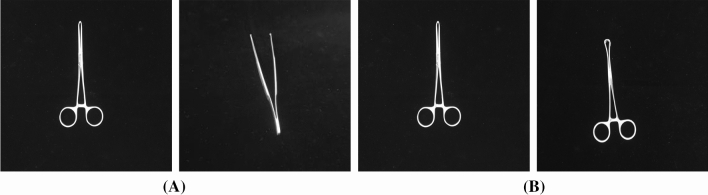
The contrast between coarse-grained classes and fine-grained classes.** a** coarse-grained classes: Alice forceps and Tissue tweezers;** b** fine-grained classes: Alice forceps and Appendix forceps

## Dataset

In this paper, we propose a new dataset called surgical instrument dataset (SID19) about the surgical instruments for the inventory work. To our knowledge, it is the first dataset to support the research of surgical instrument classification and recognition that collects surgical tools from particular surgical instrument kits of the most common surgeries. Existing works revolving around surgical instruments, i.e., the dataset of NeuroSurgicalTools [4], mainly focus on real-time tool detection, segmentation and tracking. All of these relevant datasets are proposed to provide a more precise operation understanding for doctors. Different from previous works, SID19 is introduced for the classification of inventory work and has two unique attributes. The one is various surgical instruments are collected from several certain surgical tool kits for the corresponding common surgeries. And the other is that the dataset is collected in an individual view for every tool to ensure accurate classification results under different views. There are majority of fine-grained categories in the proposed dataset which are difficult to distinguish, and the task is regarded as a medical task where high precision should be guaranteed. Hence, each image in this dataset only contains one surgical instrument object. In order to support the research of surgical instrument and present our sample library more clearly, we will publish SID19 on GitHub platform soon.

Generally speaking, one operation corresponds to one surgical instrument kit. And in fact, some surgical instrument kits contain a lot of the same surgical instruments, such as appendectomy kit and cholecystectomy kit. Therefore, based on the three most common operations including appendectomy, cholecystectomy and cesarean section, we introduce a new dataset, SID19, to collect the images of surgical tools in the three corresponding kits. Concretely, surgical instrument classes in SID19 include Abdominal wall hook, Alice forceps, Appendix forceps, Attraction tube, Bending plate, Curved tip surgical scissors, Dressing tweezers, Elbow hemostatic forceps, Integrated tissue scissors, Intestinal tract, Needle holder, No.4 tool holder, Oval forceps, Pad towel forceps, S deep pull hook, Straight hemostatic forceps, Straight tip surgical scissors, Tissue hook and Tissue tweezers.

SID19 consists of 19 classes with 3,800 images. And each class in this dataset contains 200 images. Note that, the dataset not only contains coarse-grained classes easy to identify, but also contains fine-grained classes that are difficult to differentiate. For example, Alice forceps and Tissue tweezers belong to coarse-grained classes, but Alice forceps and Appendix forceps belong to fine-grained ones as shown in Fig. 5. Note that, we generate the dataset in the daytime and night with powerful lights to simulate the circumstance of an operating room. When collecting images, each surgical instrument is placed on a black light-absorbing cloth with various postures. Especially, as to forceps and scissors classes, we adopt two strategies including open state and closed state to collect the images. Furthermore, the collecting strategy about different angles is adopted when capturing images, which is of great significance because some surgical instruments are identical in the main view but belong to different fine-grained classes, such as Straight hemostatic forceps and Elbow hemostatic forceps. Thus, it is essential to obtain their images in a side view with a specific angle. In the procedure of collecting data, we take an angle between 30 and 60 to collect images in a side view. Above all, all images in SID19 are collected during different time slots, exhibition states, postures and views. The collection environment is a unified workbench with a black light-absorbing cloth. Besides, all images are obtained with a camera in the same resolution of $3456 \times 3456$. In a word, the same shooting environment and different shooting requirements are implemented to guarantee the unity and variety of the dataset.

## Experimental results

### Implementation details

We conduct the experiments on the new proposed surgical instrument dataset, SID19, in which there are 19 classes and 200 images for each class. The ratio of training set to testing set is three to two. We use data sharding for distributed training on SID19, evenly partitioning the data across GPUs. In the data processing stage, the images are RGB-normalized via mean/standard-deviation rescaling. The size of input images is resized to $256\times 256$ for both training and testing. And then, a random resized crop is conducted for each image to get a $224\times 224$ size. Furthermore, random horizontal flip and vertical flip are employed in the training and testing stage. Besides, we train on SID19 for 50 epochs, and the default batch size is set to 64. The base learning rate is set to 0.01 (0.1 for VGGNet [37]), which decays by 10 in half and three-quarters of 50 epochs. The parameter cardinality is set to 32 for generating 32 group-wise enhanced attention maps because of the fixed optimal structure of ResNeXt50 [43]. And the reduction ratio *r* is set to 16. Specially, we employ the CBAM layer after $conv4\_6$ and $conv5\_3$ to generate the enhanced feature maps ${{\hat{\mathbf {F}}}^{3}}$ and ${{\hat{\mathbf {F}}}}^{4}$ for multi-scale learning in ResNet50. The sizes of two enhanced feature maps are $28\times 28\times 1024$ and $14\times 14\times 2028$. Then, there are two different *fc* layers to generate the corresponding prediction distributions. We adopt top-1 accuracy as the evaluation criterion and the loss is measured by using the cross-entropy function. All experiments are implemented based on Python 3.6 and PyTorch framework.

**Table 2:** Performance of ResNet50 and SKA-ResNet50 as a function of batch size

Batch_size	16	32	64	128
ResNet50 top-1 acc (%)	93.026	94.817	95.014	95.313
SKA-ResNet50 top-1 acc (%)	95.925	97.703	97.754	97.758

**Table 3:** Performance of ResNet50 and SKA-ResNet50 as a function of scale of input data

Scale	112	224	336	448
ResNet50 top-1 acc (%)	91.341	95.014	95.483	95.804
SKA-ResNet50 top-1 acc (%)	95.187	97.703	97.739	97.813

### Ablation study

**Batch Size** The number of batch size controls the number of mini-batch in a training and testing iteration. As batch size is one of the most vital factors which has a great influence on the weights update and generalization performance of models, an appropriate batch size is essential. In the experiments, we adopt 16, 32, 64 and 128 as the size of a mini-batch, respectively. From Table 2, it is concluded that with the increase in batch size, the performance of SKA-ResNet and ResNet shows a trend of increasing first and then tending to be stable. Through the experimental results, we recommend the batch size to be 32 or 64 so that there will be an accurate result without occupying too much memory space. In subsequent experiments, we use 32 as the batch size by default.

**Scale** The scale of input data is resized generally before being sent to the network and has a direct impact on the classification results. If the scale is too small, there will be serious information loss. On the contrary, the abstract level of information is not high enough and large calculations are brought. Generally, input data are resized to a scale of $224\times 224$. In the experiments, four different scales are investigated as shown in Table 3. It is concluded that the performance tends to increase gradually with the scale increasing. However, the grown of tendency is inconspicuous from the scale of 224 to 448. Therefore, the scale of input data is resized to $224 \times 224$ in the conditions without special instructions.

**Effectiveness of MSL** Table 4 reveals the effectiveness of our multi-scale learning along with the proposed regularizer utilizing different constraint strategies. Specially, we express the regularizer by ’+,’ indicating which two feature maps have such constraint. There are three relationships among ${\mathbf {P}}^{3}$, ${\mathbf {P}}^{4}$ and ${\mathbf {P}}$. The strategy of ${\mathbf {P}}^{3} + {\mathbf {P}}^{4}$ means that we encourage the prediction distribution ${\mathbf {P}}^{3}$ to match with ${\mathbf {P}}^{4}$. The intention of ${\mathbf {P}} + {\mathbf {P}}^{4}$ and ${\mathbf {P}}^{3} + {\mathbf {P}}$ is similar with ${\mathbf {P}}^{3} + {\mathbf {P}}^{4}$. In the experiments, we also combine the three strategies forming the other three strategies. As shown in Table 4, the strategy combining all the individual strategy of ${\mathbf {P}}^{3} + {\mathbf {P}}^{4}, {\mathbf {P}} + {\mathbf {P}}^{4}, {\mathbf {P}}^{3} + {\mathbf {P}}$ can achieve the best performance. We also obtain that the effectiveness of individual strategy ${\mathbf {P}}^{3} + {\mathbf {P}}^{4}$ can also achieve a better performance comparing with ${\mathbf {P}} + {\mathbf {P}}^{4}$ and ${\mathbf {P}}^{3} + {\mathbf {P}}$. ${\mathbf {P}}^{3}$ corresponds to *stage*3 which contains more precise location information and ${\mathbf {P}}^{4}$ is the output of *stage*4 which bears more discriminative semantic information. Exploring the relationship between two parts is more effective than the other two strategies. Besides, the results of methods with CBAM layer outperform that without CBAM layer.

**Table 4:** Ablation performance on SID19 with CBAM layer and different MSL regularizer strategies alternatively employed on ResNet-50 with SKA module

Method	top-1 acc (%)	
	w/o CBAM layer	w/ CBAM layer
w/o MSL	97.042	-
${\mathbf {P}} + {\mathbf {P}}^{4}$	96.985	97.117
${\mathbf {P}}^{3} + {\mathbf {P}}^{4}$	97.476	97.611
${\mathbf {P}}^{3} + {\mathbf {P}}$	97.002	97.183
${\mathbf {P}}^{3} + {\mathbf {P}}^{4}, {\mathbf {P}} + {\mathbf {P}}^{4}$	97.538	97.646
${\mathbf {P}}^{3} + {\mathbf {P}}^{4}, {\mathbf {P}}^{3} + {\mathbf {P}}$	97.497	97.619
${\mathbf {P}}^{3} + {\mathbf {P}}^{4}, {\mathbf {P}} + {\mathbf {P}}^{4}, {\mathbf {P}}^{3} + {\mathbf {P}}$	97.602	**97.703**

**Table 5:** Comparing our SKA-ResNet50 with ResNet50, SGE-ResNet50 and SK-ResNet50 on SID19

Method	Param.	GFLOPS	top-1 acc (%)
ResNet50 [12]	25.56M	4.122	95.014
SGE-ResNet50 [21]	25.56M	4.127	96.203
SK-ResNet50 [22]	26.15M	4.185	96.197
SKA-ResNet50(w/o MSL)	26.15M	4.195	**97.042**

**Fig. 6:**
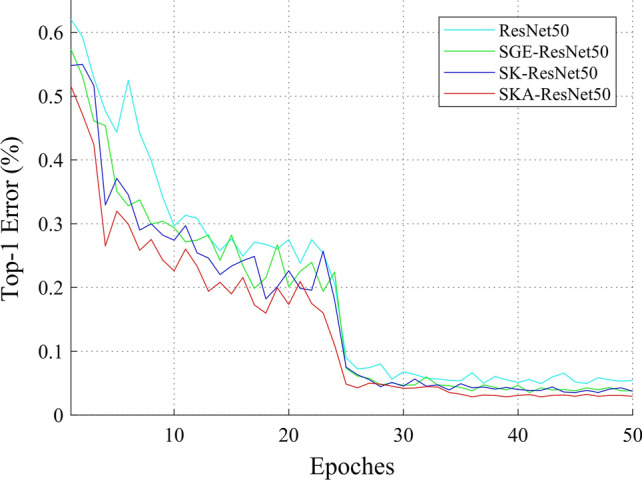
Top-1 error curves on SID19 based on ResNet50, SGE-ResNet50, SK-ResNet50 and SKA-ResNet50

**Effectiveness of SKA module** The effectiveness of our SKA module is studied in Fig. 6 and Table 5. For a fair comparison, all the methods do not use the strategy of multi-scale learning. As the core of SKA-ResNet, SKA block adds a novel selective kernel mechanism with attention to the end of the standard residual block, which further improves the accuracy of ResNet50 from 95.014% to 97.042%. Comparing with SGE module and SK module, SKA module is equivalent to equipping multi-branch adaptive kernel selection feature maps with spatial group-wise enhanced attentions, and accuracy is further boosted from 96.203% and 96.197% to 97.042%. Meanwhile, the rate of convergence of SK-ResNet50, SGE-ResNet50 and our model are obviously faster than ResNet50. Apparently, the convergence rate of the proposed model lies at a near level with the highest performance as shown in Fig. 6. Furthermore, the number of parameters and calculations about the four models are displayed in Table 5. It is concluded that our model can obtain the best performance without introducing too many parameters and calculations at almost the same time.

**Table 6:** Experimental results on SID19. All state-of-the-art methods with lightweight attention modules are displayed

Model	Param.	GFLOPS	top-1 acc (%)
ResNet [12]	25.56M	4.122	95.621
SE-ResNet [13]	28.09M	4.130	96.607
BAM-ResNet [32]	25.92M	4.205	96.622
CBAM-ResNet [42]	28.09M	4.139	96.618
SK-ResNet [22]	26.15M	4.185	96.947
SGE-ResNet [21]	25.56M	4.127	96.913
SKA-ResNet(ours)	26.15M	4.195	**97.703**

That all these methods are implemented with the proposed multi-scale regularizer

### Comparison with state-of-the-Art on SID19

**Comparison with Lightweight Attention modules** We compare our SKA-ResNet with several prevailing methods that are embedded into lightweight attention modules. In Table 6, quantitative experimental results on SID19 are exhibited. For a fair comparison, all displayed methods are implemented with the proposed multi-scale regularizer based on a unified ResNet50 backbone. And all of the attention modules are employed after the last BatchNorm layer within every bottleneck in ResNet50. As shown in Table 6, it is observed that the proposed SKA-ResNet with SKA module for generating attention maps with adaptive kernel selection mechanism achieves the best overall performance against the prevalent attention modules. SGE-ResNet and SK-ResNet achieve a close accuracy of around 96.9% by leveraging spatial group-wise enhanced attention and adaptive kernel selection mechanism, respectively. However, our SKA-ResNet model embeds the multi-branch SGE attentions into adaptive kernel selection module, which outperforms SGE-ResNet and SK-ResNet about 0.7%. Other mainstream methods based on attention modules, such as SE-ResNet, BAM-ResNet, CBAM-ResNet, can achieve an accuracy of around 96.6%, which is lower than SGE-ResNet and SK-ResNet with a margin of 0.35%. In particular, our method has fewer parameters and calculations compared with SE-ResNet and CBAM-ResNet. Importantly, there is no great amount of parameters and calculations are introduced in our proposed model in contrast to other methods.

**Table 7:** Experimental results based on specific attention-based methods for FGVC

Method	Backbone	1-Stage	top-1 acc (%)
FCAN [28]	ResNet50	$\checkmark$	97.227
RA-CNN [9]	VGG-19		96.932
MA-CNN [48]	VGG-19	$\checkmark$	96.859
MAMC [38]	ResNet50	$\checkmark$	96.883
DT-RAM [23]	ResNet50		96.765
DFL-CNN [41]	ResNet50	$\checkmark$	97.197
NTS-Net [44]	ResNet50	$\checkmark$	97.314
SKA-ResNet(ours)	ResNet50	$\checkmark$	**97.703**

The third column indicates whether the method is trained and tested in one stage or not

**Table 8:** Experimental results based on other methods for FGVC

Method	Backbone	Top-1 acc (%)
	VGG-D	94.978
Bilinear Pooling [25]	ResNet50	96.574
Compact Bilinear Pooling [10]	ResNet50	96.697
iSQRT-COV [20]	ResNet50	97.185
	ResNet50	97.220
Cross-X [29]	SENet	97.213
SGENeXt(ours)	ResNet50	**97.703**

The displayed methods include high-order statistics learning and multi-scale feature relationship learning

**Comparison with Attention-based Methods for FGVC** In Table 7, we compare our SKA-ResNet to attention-based methods for FGVC on SID19. All the displayed models are weakly supervised and introduce additional attention networks to learn the representation of discriminative regions. The attribute column “1-Stage” in table indicates that these methods can be trained and tested end-to-end in only one stage. From the presented statistics, our method achieves the state-of-the-art performance on SID19, even though RA-CNN and NTS-Net employ recurrent crops and multi-scale crops, respectively. MA-CNN and MAMC attain a similar result around 96.8% due to the introduction of multiple feature maps. However, compared to our method, the classification performance is reduced by around 0.9%. Focusing on one discriminative region, RA-CNN obtains a better result than MA-CNN and MAMC, but our SKA-ResNet can further obtain 0.8% relative improvement. Furthermore, we can also observe that our method achieves the best performance comparing with other one-stage methods. More than anything, compared with these attention-based methods introducing additional attention networks, our method only introduces few computational burdens and the number of parameters with the best performance due to the lightweight attention module and multi-scale regularizer.

**Comparison with Other Methods for FGVC** There are other methods introduced for solving FGVC, such as high-order statistics learning and multi-scale feature relationship learning. As is shown in Table 8, non-attention-based methods for FGVC are implemented on SID19. For a fair comparison, all of the displayed models possess the same unified ResNet50 as the backbone. Especially, we also implement the bilinear pooling and cross-X learning on VGG-D and SENet, respectively. From Table 8, we observe that iSQRT-COV, a high order statistic method based on bilinear pooling and compact bilinear pooling, achieves an accuracy of 97.185%, which outperforms the two methods by about 0.5%. As to multi-scale feature relationship learning, two models based on cross-X learning achieve a close performance around 97.2%, decreasing by 0.5% of our method. It is concluded that all of the methods in Table 8 hold lower accuracy than the proposed method. The improvement indicates the effectiveness of the two main components of SKA-ResNet.

**Table 9:** Comparison of our approach to recent results on four standard FGVC datasets: CUB-200-2011, Stanford Cars, Stanford Dogs and FGVC-Aircraft

Method	Accuracy(%)			
	Birds	Cars	Dogs	Aircraft
ResNet-50 [12]	84.5	92.9	88.1	90.3
FCAN [28]	84.7	93.1	88.9	–
RA-CNN [9]	85.3	92.5	87.3	88.2
MA-CNN [48]	86.5	92.8	–	89.9
MAMC [38]	86.2	93.0	84.8	–
DFL-CNN [41]	87.4	93.1	–	91.7
NTS-Net [44]	87.5	93.9	–	91.4
B-CNN [25]	84.1	91.3	–	84.1
iSQRT-COV [20]	88.1	92.8	–	90.0
Cross-X [29]	87.7	94.6	88.9	92.6
SKA-ResNet(ours)	**88.3**	**94.7**	**89.1**	**92.6**

### Comparison with state-of-the-art on FGVC datasets

The comparison results on four challenging FGVC datasets including CUB-200-2011 (Birds), Stanford Cars (Cars), Stanford Dogs (Dogs) and FGVC Aircraft (Aircraft) are reported in Table 9. Considering that we do not use any bounding box/part annotations in all our experiments, some of the compared approaches depending on bounding box/part annotations are not presented in parentheses for direct comparisons. From Table 9, we can see that our approach achieves state-of-the-art or comparable results on four datasets. In particular, we obtain the best performance in terms of accuracy (as highlighed by the bold values in Table 9 on CUB-200-2011, Stanford Cars, Stanford Dogs. Meanwhile, the result of our method on Aircraft is comparable with the state-of-the-art methods.

Experimental results are grouped into three parts in Table 9. As a strong baseline, the results of ResNet-50 by itself are shown in the first part, while our SKA-ResNet outperforms it on all datasets. The results of a certain number of attention-based methods are presented in the second part. Compared with these approaches focusing on constructing complex attention networks for discriminative regions, our approach embeds a lightweight adaptive kernel selection module with SGE attentions into the residual blocks to strengthen the intermediate feature maps. It is clearly summarized that we achieve state-of-the-art performance on four datasets, and it is worth noting that our approach does not introduce too many parameters and calculations. Furthermore, we report the results of other FGVC methods in the third part. We find that the performance of our approach outperforms that of iSQRT-COV and cross-X learning, which are state-of-the-art feature relationship learning methods. However, the optimization of our method is much easier due to the embedding SKA module in the feature extractor.

**Fig. 7:**
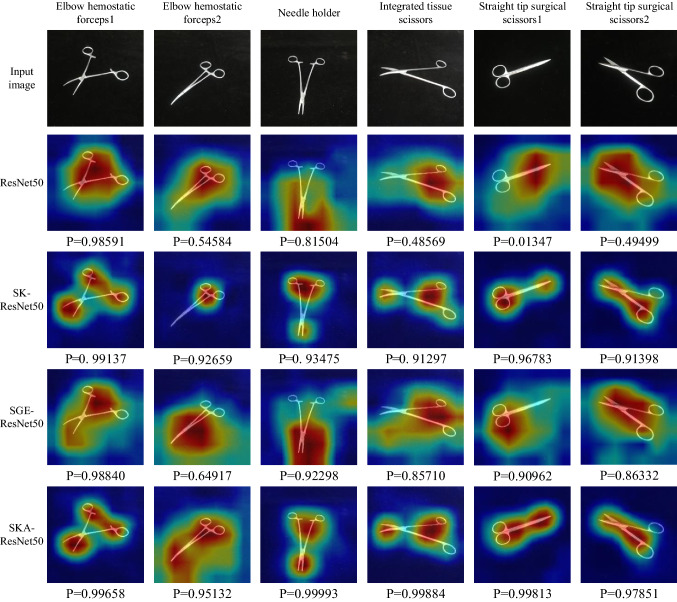
Visualization results of ResNet50, SK-ResNet50, SGE-ResNet50 and SKA-ResNet50. The activation map is calculated for the last convolutional outputs. The ground-truth label is shown on the top of each input image and P denotes the softmax score of each network for the ground-truth class

### Network visualization

Fig. 7 depicts the resized activation maps of six images from SID19 (including Elbow hemostatic forceps1, Elbow hemostatic forceps2, Needle holder, Integrated tissue scissors, Straight surgical scissors1 and Straight surgical scissors2) based on four different models including ResNet50, SGE-ResNet50, SK-ResNet50 and SKA-ResNet50. The activation map of a certain layer usually strongly emphasizes discriminative regions of the input image. It is intuitively understood that how the network works in a certain layer by observing the highlighted regions in the activation map. We obtain activation maps by using the same method [49]. As the probability for target class, the softmax scores are displayed below the corresponding activation maps for the qualitative analysis.

From Fig. 7, we can obviously observe that SKA-ResNet50 covers more complete object regions than the other three models. Meanwhile, the discriminative regions in activation maps based on SKA-ResNet50 are even brighter. In the aspect of softmax scores, SKA-ResNet50 takes a more precise probability than other networks, which is harmonious with the activation maps.

## Conclusion

In this paper, we take a series of research works on surgical instrument image classification. Firstly, the work for surgical instrument image classification is proposed to assist medical staff with the inventory work of medical instrument under the background of reducing the risk of surgical instrument loss after surgery. Secondly, we collect the first surgical instrument dataset called SID19 based on the three most common surgeries to support the research. More importantly, we propose a novel attention-based model called SKA-ResNet with lightweight SKA modules to strengthen informative feature maps of discriminative regions and a multi-scale regularizer to exploit the relationships between different feature maps. And a myriad of state-of-the-art classification models are implemented on the proposed dataset and four challenging FGVC datasets. Experimental results show that our approach achieves state-of-the-art performance, which is enough to be the theoretical basis for inventory work. Ablation studies further prove the effectiveness of the components in SKA-ResNet.

In the future work, it is planned to be processed into the object detection task of surgical instruments when solving the inventory work of surgical instruments. Specifically, firstly, object annotation is performed on the existing surgical instrument dataset and combined with the fine-grained image classification algorithm proposed in this paper and object detection algorithm, the object detection of single object image is initially realized. Then, the object detection task of multiple similar surgical instruments can be realized step by step, which further serves the inventory work of surgical instruments.

## References

[1] Agatonovickustrin S, Beresford R (2000). Basic concepts of artificial neural network (ann) modeling and its application in pharmaceutical research. J Pharm Biomed Anal.

[2] Apostolopoulos ID, MT (2020) Covid-19: automatic detection from x-ray images utilizing transfer learning with convolutional neural networks. Phys Eng Sci Med 43(2): 635–640 10.1007/s13246-020-00865-4 PMC7118364 32524445

[3] Balakrishnan G, Zhao A, Sabuncu MR, Dalca AV, Guttag JV (2018) An unsupervised learning model for deformable medical image registration. IEEE Conference on Computer Vision and Pattern Recognition (CVPR) pp. 9252–9260

[4] Bouget D, Benenson R, Omran M, Riffaud L, Schiele B, Jannin P (2015) Detecting surgical tools by modelling local appearance and global shape. IEEE Trans Med Imaging 34(12): 2603–2617 10.1109/TMI.2015.2450831 26625340

[5] Cai Z, Fan Q, Feris RS, Vasconcelos N (2016) A unified multi-scale deep convolutional neural network for fast object detection. Proceedings of the European Conference on Computer Vision (ECCV) pp. 354–370

[6] Caraiman S, Zvoristeanu O, Burlacu A, Herghelegiu P (2019) Stereo vision based sensory substitution for the visually impaired. Sensors 19(12): 2771–2788 10.3390/s19122771 PMC6630569 31226796

[7] Chilamkurthy S, Ghosh R, Tanamala S, Biviji M, Campeau NG, Venugopal VK, Mahajan V, Rao P, Warier P (2018) Deep learning algorithms for detection of critical findings in head ct scans: a retrospective study. The Lancet 392, (10162): 2388–2396 10.1016/S0140-6736(18)31645-3 30318264

[8] Filho PPR, Barros ACDS, Ramalho GLB, Pereira CR, Papa JP, De Albuquerque VHC, Tavares JMRS (2019) Automated recognition of lung diseases in ct images based on the optimum-path forest classifier. Neural Comput Appl, 31(2): 901–914 (2019)

[9] Fu J, Zheng H, Mei T (2017) Look closer to see better: Recurrent attention convolutional neural network for fine-grained image recognition. IEEE Conference on Computer Vision and Pattern Recognition (CVPR) pp. 4438–4446

[10] Gao Y, Beijbom O, Zhang N, Darrell T (2016) Compact bilinear pooling. IEEE Conference on Computer Vision and Pattern Recognition (CVPR) pp. 317–326

[11] Garciaperazaherrera LC, Li W, Gruijthuijsen C, Devreker A, Attilakos G, Deprest J, Poorten EV, Stoyanov D, Vercauteren T, Ourselin S (2016) Real-time segmentation of non-rigid surgical tools based on deep learning and tracking. Lect Notes Comput Sci, 10170: 84–95

[12] He K, Zhang X, Ren S, Sun J (2016) Deep residual learning for image recognition. IEEE Conference on Computer Vision and Pattern Recognition (CVPR) pp. 770–778

[13] Hu J, Shen L, Sun G (2018) Squeeze-and-excitation networks. IEEE Conference on Computer Vision and Pattern Recognition (CVPR) pp. 7132–7141

[14] Huang S, Xu Z, Tao D, Zhang Y (2016) Part-stacked cnn for fine-grained visual categorization. IEEE Conference on Computer Vision and Pattern Recognition (CVPR) pp. 1173–1182

[15] Jeganathan VE, Shah S (2009) Robotic technology in ophthalmic surgery. Curr Opin Ophthalmol 21: 75–80 10.1097/ICU.0b013e328333371d 19829111

[16] Kalan S, Chauhan S, Coelho RF, Orvieto MA, Camacho I, Palmer KJ, Patel VR (2010) History of robotic surgery. JRobotic Surg 4(3): 141–147 10.1007/s11701-010-0202-2 27638753

[17] King BF (2018) Artificial intelligence and radiology: What will the future hold?. J Am College Radiol 15(3): 501–503 10.1016/j.jacr.2017.11.017 29371088

[18] Kong T, Yao A, Chen Y, Sun F (2016) Hypernet: Towards accurate region proposal generation and joint object detection. IEEE Conference on Computer Vision and Pattern Recognition (CVPR) pp. 845–853

[19] Li H, Giger ML, Huynh BQ, Antropova N (2017) Deep learning in breast cancer risk assessment: evaluation of convolutional neural networks on a clinical dataset of full-field digital mammograms. Journal of medical imaging 4(4):041304 10.1117/1.JMI.4.4.041304 PMC5596198 28924576

[20] Li P, Xie J, Wang Q, Gao Z (2018) Towards faster training of global covariance pooling networks by iterative matrix square root normalization. IEEE Conference on Computer Vision and Pattern Recognition (CVPR) pp. 947–955

[21] Li X, Hu X, Yang J (2019) Spatial group-wise enhance: Improving semantic feature learning in convolutional networks. arXiv preprint arXiv: Computer Vision and Pattern Recognition

[22] Li X, Wang W, Hu X, Yang J (2019) Selective kernel networks. IEEE Conference on Computer Vision and Pattern Recognition (CVPR) pp. 510–519

[23] Li Z, Yang Y, Liu X, Zhou F, Wen S, Xu W (2017) Dynamic computational time for visual attention. IEEE International Conference on Computer Vision (ICCV) pp. 1199–1209

[24] Lin D, Shen X, Lu C, Jia J (2015) Deep lac: deep localization, alignment and classification for fine-grained recognition. IEEE Conference on Computer Vision and Pattern Recognition (CVPR) pp. 1666–1674

[25] Lin T, Roychowdhury A, Maji S (2015) Bilinear cnn models for fine-grained visual recognition. IEEE International Conference on Computer Vision (ICCV) pp. 1449–1457

[26] Lin TY, Dollár P, Girshick R, He K, Hariharan B, Belongie S (2017) Feature pyramid networks for object detection. IEEE Conference on Computer Vision and Pattern Recognition (CVPR) pp. 2117–2125

[27] Liu W, Anguelov D, Erhan D, Szegedy C, Berg AC (2016) Ssd: single shot multibox detector. Proceedings of the European Conference on Computer Vision (ECCV) pp. 21–37

[28] Liu X, Xia T, Wang J, Yang Y, Zhou F, Lin Y (2016) Fully convolutional attention networks for fine-grained recognition. arXiv preprint arXiv:1603.06765

[29] Luo W, Yang X, Mo X, Lu Y, Davis LS, Li J, Yang J, Lim S (2019) Cross-x learning for fine-grained visual categorization. IEEE International Conference on Computer Vision (ICCV) pp. 8242–8251

[30] Milletari F, Navab N, Ahmadi S (2016) V-net: fully convolutional neural networks for volumetric medical image segmentation. 2016 Fourth International Conference on 3D Vision (3DV) pp. 565–571

[31] Pakhomov D, Premachandran V, Allan M, Azizian M, Navab N (2017) Deep residual learning for instrument segmentation in robotic surgery. arXiv preprint arXiv: Computer Vision and Pattern Recognition

[32] Park J, Woo S, Lee J, Kweon IS (2018) Bam: Bottleneck attention module. arXiv preprint arXiv: Computer Vision and Pattern Recognition

[33] Prati A, Shan C, Wang KI (2019) Sensors, vision and networks: From video surveillance to activity recognition and health monitoring. J Ambient Intell Smart Environ 11(1): 5–22

[34] Roth HR, Lu L, Liu J, Yao J, Seff A, Cherry KM, Kim L, Summers RM (2016) Improving computer-aided detection using convolutional neural networks and random view aggregation. IEEE Trans Med Imaging 35(5): 1170–1181 10.1109/TMI.2015.2482920 PMC7340334 26441412

[35] Sanchezgarcia M, Martinezcantin R, Guerrero JJ (2020) Semantic and structural image segmentation for prosthetic vision. PLOS ONE, 15(1) 10.1371/journal.pone.0227677 PMC6988941 31995568

[36] Sekaran K, Chandana P, Krishna NM, Kadry S (2019) Deep learning convolutional neural network (cnn) with gaussian mixture model for predicting pancreatic cancer. Multimedia Tools and Applications pp. 1–15

[37] Simonyan K, Zisserman A (2014) Very deep convolutional networks for large-scale image recognition. arXiv preprint arXiv:1409.1556

[38] Sun M, Yuan Y, Zhou F, Ding E (2018) Multi-attention multi-class constraint for fine-grained image recognition. Proceedings of the European Conference on Computer Vision (ECCV) pp. 805–821

[39] Suo Q, Ma F, Yuan Y, Huai M, Zhong W, Zhang A, Gao J (2017) Personalized disease prediction using a cnn-based similarity learning method. IEEE International Conference on Bioinformatics and Biomedicine pp. 811–816

[40] Wang S, Kang B, Ma J, Zeng X, Xiao M, Guo J, Cai M, Yang J, Li Y, Meng X, et al (2021) A deep learning algorithm using ct images to screen for corona virus disease (covid-19). European radiol pp. 1–9 10.1007/s00330-021-07715-1 PMC7904034 33629156

[41] Wang Y, Morariu VI, Davis LS (2018) Learning a discriminative filter bank within a cnn for fine-grained recognition. IEEE Conference on Computer Vision and Pattern Recognition (CVPR) pp. 4148–4157

[42] Woo S, Park J, Lee J, Kweon IS (2018) Cbam: convolutional block attention module. Proceedings of the European Conference on Computer Vision (ECCV) pp. 3–19

[43] Xie S, Girshick R, Dollar P, Tu Z, He K (2017) Aggregated residual transformations for deep neural networks. IEEE Conference on Computer Vision and Pattern Recognition (CVPR) pp. 5987–5995

[44] Yang Z, Luo T, Wang D, Hu Z, Gao J, Wang L (2018) Learning to navigate for fine-grained classification. Proceedings of the European Conference on Computer Vision (ECCV) pp. 420–435

[45] Zhang N, Donahue J, Girshick R, Darrell T (2014) Part-based r-cnns for fine-grained category detection. Proceedings of the European Conference on Computer Vision (ECCV) pp. 834–849

[46] Zhao R, Yan R, Chen Z, Mao K, Wang P, Gao RX (2019) Deep learning and its applications to machine health monitoring. Mech Syst Signal Processing 115: 213–237

[47] Zhao Z, Chen Z, Voros S, Cheng X (2019) Real-time tracking of surgical instruments based on spatio-temporal context and deep learning. Comput Assisted Surg 24(sup1): 20–29 10.1080/24699322.2018.1560097 30760050

[48] Zheng H, Fu J, Tao M, Luo J (2017) Learning multi-attention convolutional neural network for fine-grained image recognition. IEEE International Conference on Computer Vision (ICCV) pp. 5219–5227

[49] Zhou B, Khosla A, Lapedriza A, Oliva A, Torralba A (2016) Learning deep features for discriminative localization. IEEE Conference on Computer Vision and Pattern Recognition (CVPR) pp. 2921–2929

[50] Zhou Y, He L, Huang Y, Chen S, Wu P, Ye W, Liu Z, Liang C (2017) Ct-based radiomics signature: a potential biomarker for preoperative prediction of early recurrence in hepatocellular carcinoma. Abdominal Radiology 42(6): 1695–1704 10.1007/s00261-017-1072-0 28180924

